# Safety and Protective Effectiveness of Two Strains of *Lactobacillus* with Probiotic Features in an Experimental Model of Salmonellosis

**DOI:** 10.3390/ijerph110908755

**Published:** 2014-08-26

**Authors:** Raphael S. Steinberg, Lilian C. S. Silva, Tássia C. Souza, Maurício T. Lima, Nayara L. G. de Oliveira, Leda Q. Vieira, Rosa M. E. Arantes, Anderson Miyoshi, Jacques R. Nicoli, Elisabeth Neumann, Álvaro C. Nunes

**Affiliations:** 1 Department of General Biology, Institute of Biological Sciences, Federal University of Minas Gerais, Av. Antonio Carlos 6627, 31270-901, Belo Horizonte, Brazil; E-Mails: raphael.steinberg.ds@gmail.com (R.S.S.); lilian_cristina@ymail.com (L.C.S.S.); miyoshi@icb.ufmg.br (A.M.); 2 Department of Microbiology, Institute of Biological Sciences, Federal University of Minas Gerais, Av. Antonio Carlos 6627, 31270-901, Belo Horizonte, Brazil; E-Mails: tassiacosta@yahoo.com.br (T.C.S.); maurili15@hotmail.com (M.T.L.); nayaralane@hotmail.com (M.L.G.O.); jnicoli@icb.ufmg.br (J.R.N.); eneumann@icb.ufmg.br (E.N.); 3 Department of Biochemistry and Immunology, Institute of Biological Sciences, Federal University of Minas Gerais, Av. Antonio Carlos 6627, 31270-901, Belo Horizonte, Brazil; E-Mail: lqvieira@icb.ufmg.br; 4 Department of General Pathology, Institute of Biological Sciences, Federal University of Minas Gerais, Av. Antonio Carlos 6627, 31270-901, Belo Horizonte, Brazil; E-Mail: rosa@icb.ufmg.br

**Keywords:** salmonellosis, mouse experimental model, immunomodulation, *Lactobacillus*, cattle

## Abstract

Two strains of *Lactobacillus*, previously isolated from bovine faeces and tested *in vitro* for properties desired in probiotics, were evaluated for their *in vivo* effectiveness in protecting against experimental salmonellosis. *L. salivarius* L38 and *L. acidophilus* L36 previously demonstrated the ability to successfully colonize the gastrointestinal tract of germ-free mice and stimulate the immune system associated with the intestinal mucosa. L38- or L36-feeding showed no detrimental effect on the general health indicators and did not induce changes in normal architecture of liver and small intestine, indicating that the use of these strains is apparently safe. In control animals fed L38 strain, several cytokines had augmented mRNA levels that can be associated with a homeostatic state of intestinal mucosa, while L36 had less diverse regulation. IgA production and secretion in the intestinal lumen induced by infection was abrogated by pretreating with both lactobacilli. In addition, liver and small intestine histological scores and, translocation of *Salmonella* cells to liver and spleen, indicated that these strains did not confer protection against the infection. So, the IL-12:IL-18➔IFN-γ axis, essential for an effective immune response against *Salmonella*, was not favored with L38 or L36 strains. However, increased expression of IL-10 in different portions of the gastrointestinal tract of L38-fed animals is indicative of anti-inflammatory effect to be explored furthermore.

## 1. Introduction

Probiotics, “live microorganisms, which when consumed in adequate amounts, confer a health effect on the host” [1], are receiving special attention from farmers who are looking for alternatives to the use of traditional antibiotics as growth promoters. Indeed, injudicious prophylactic use of antibiotics has been banned by governmental regulatory agencies, in the European Union by the Regulation EC 1831/2003 of the European Parliament and of the Council of 22 September 2003 on additives for use in animal nutrition [2] and in the USA by the U.S. FDA CVM GFI #209 [3] and #213 2012 [4], because of the danger of multidrug-resistant bacteria selection. It was already shown that immunomodulation by several probiotics maintains the immune system primed to permit a faster and effective response in the case of infections [5].

Lactic Acid Bacteria (LAB) isolated from animal and human intestines have acquired the “Generally recognized as safe” (GRAS) status after a long history of use as food supplements, fermenting natural products, starter cultures to make foods, and probiotics [6]. In most animals, these beneficial gut bacteria interact actively with other microorganisms belonging to the indigenous microbiota and with transient pathogens, inhibiting the installation of exogenous or uncontrolled multiplication of the commensals [5]. It has been demonstrated that probiotics can replace conventional growth promoters such as antibiotics, especially in newly born animals [7].

Poultry are the most widely studied livestock with respect to the use of probiotics. Selected strains of *Lactobacillus* bearing probiotic properties are used to control *Salmonella* infection in the intestinal tract of chickens [8]. Another approach is to inoculate one-day-old chicks with cultures of the intestinal microbiota from a healthy chicken in order to establish a healthy, normal flora, which helps control salmonellosis. However, this approach is hard to reproduce from one animal batch to another due to differences in the organisms composing the preparations. Direct-fed microbials (DFM), an approach employed in cattle, significantly reduced the probability of new infections with *Salmonella* and fecal shedding of *Escherichia coli* O157:H7 [9,10].

Probiotics exert their beneficial effects by three action lines: direct antimicrobial activity, enhancement of intestinal barrier function and, local and systemic immunomodulation [11,12]. Some probiotic bacteria are able to increase the number of producing IgA (immunoglobulin type A) cells in the lamina propria. This increase improves the responses triggered by oral vaccines [13,14,15]. Furthermore, probiotics act in local and systemic regulation of the immune system particularly by stabilizing and maintaining of balance between pro-inflammatory and regulatory molecules mostly secreted by T-cell populations (T_H_1/T_H_2/T_H_17/T_reg_) [16,17,18]. Therefore, in this context intake of probiotics is a safe and non-drug approach to combat pathogenic microbes, mainly via modulation of the immune system [19].

The safety and immunomodulatory effects of some probiotic strains have been investigated using laboratory animal models [1]. Mice challenged with *Salmonella enterica* serovar Typhimurium represent a good model for the study of the protective and therapeutic effects of probiotic bacteria against intracellular enteropathogens [19]. Probiotics, such as *Saccharomyces boulardii*, *S. cerevisiae*, *Escherichia coli* EMO, *Bifidobacterium longum*, *B. lactis*, *B. bifidum*, *Enterococcus faecium* and, *Lactobacillus bulgaricus* confer a protective effect to challenged mice [20,21,22,23]. The protective effect of some probiotic strains is related to stimulation of local production of sIgA, as well as increased activity of immune cells associated with the gut [24,25].

Nowadays, probiotic lactobacilli are used to improve the general health indicators in cattle rearing [26,27]. Side effects of using probiotics to increase livestock production are improved quality and biological value of the final commercialized products, and thus have a positive impact on human health. *L. salivarius* L38 and *L. acidophilus* L36 were recently isolated from bovine feces, successfully colonized the gastrointestinal tract of germ-free animals, and stimulated expression of different cytokine profiles in the intestinal mucosa [28]. Herein, we characterize the induced immune responses of these two isolated bovine-based microbials in a mouse model of salmonellosis. This is the first study reporting on the immunomodulation caused by bovine lactobacilli in a conventional mouse model bearing complex established microbiota. The model presented here may help future research on the evaluation of the use of probiotics in cattle rearing.

## 2. Experimental Section

### 2.1. Animals

Conventional 4- to 5-week-old Swiss NIH mice from both sexes (Taconic, Germantown, NY, USA) were used in this study. The animals were housed in plastic mini-isolators in ventilated racks (Alesco, São Paulo, Brazil), maintained in the animal house under controlled lighting (12:12 h, light:dark), and handled according to the standards outlined in the “Colegio Brasileiro de Experimentação Animal” rules [29]. The animals fed a commercial diet for rodents (Nuvital, Curitiba, Brazil) *ad libitum*. The Institutional Ethics Committee on Animal Experimentation (CETEA/UFMG) approved all experiments under agreement number 203/09 and 96/11.

### 2.2. Bacteria

*Lactobacillus acidophilus* L36 and *Lactobacillus salivarius* L38, both strains of cattle origin, were isolated, identified at the species level, and characterized *in vitro* for a number of probiotic features (acid and bile tolerance, cell surface hydrophobicity, antagonism against selected Gram-positive and Gram-negative pathogens, hydrogen peroxide production, and antimicrobial susceptibility) in a previous work of our team (unpublished data). The *Salmonella* strain of human origin was obtained in pure culture form from Fundação Ezequiel Dias (FUNED, Belo Horizonte, Brazil) and the identification as *Salmonella enterica* serovar Typhimurium was confirmed by Institut Pasteur (Paris, France). The isolated bacterium was stored at ‒70 °C in medium containing 20% glycerol.

### 2.3. Pre-treatment of Mice with Lactobacillus Strains

The strains of *Lactobacillus* were grown in de Man, Rogosa and Sharp (MRS) broth (Accumedia, Neogen Corp., Lansing, MI, USA) for 18 h at 37 °C in an anaerobic chamber (Forma Scientific Co., Marietta, OH, USA) containing an atmosphere of 85% N_2_, 10% H_2_, and 5% CO_2_. The activated culture was centrifuged at 2000 × g at 4 °C, washed twice with saline, and suspended in saline in order to obtain 9.0 log colony forming units (cfu)/mL. Daily doses of 0.1 mL of the suspension were administered to mice by gavage, during 10 days before the challenge with the pathogenic bacteria, and then throughout the remaining experimental period. The control groups were treated with 0.9% saline according to the same schedule as the corresponding experimental groups. All the groups comprised five mice, regardless of sex, and experiments were performed in duplicate, totaling ten animals per group.

### 2.4. Pathogen Challenge

*Salmonella enterica* ser. Typhimurium was grown in liquid brain heart infusion (BHI) medium (Accumedia) for 18 h at 37 °C. The culture was centrifuged at 2000 g at 4 °C, washed twice with saline, and suspended in saline. Mice were challenged by gavage with 0.1 mL of the bacterial suspension containing about 10^7^ cfu. In 9th day after challenge, mice were sacrificed by cervical dislocation. Animal weight variation (g) was measured as the difference in weight at the day of sacrifice relative to the weight on the first day of treatment with the lactobacilli. Spleen and liver size indexes were expressed as the spleen or liver weight (mg) divided by body weight (g) at the day of sacrifice. Fragments with 1–2 cm length from portions of the intestinal tract—proximal (PS), middle (MS), and distal (DS) segments of the small intestine, caecum, and colon—of these animals were removed and stored in RNA later (Ambion, Austin, TX, USA) and frozen at −20 °C for posterior extraction of total RNA.

### 2.5. Intestinal Secretory Immunoglobulin Type A (sIgA)

The small intestine of mice was removed and the contents were withdrawn, weighed, and suspended in PBS at 500 mg of intestinal contents per 2.0 mL PBS supplemented with a protease inhibitor cocktail containing aprotinin, leupeptin, pepstatin A, bestatin, E-64 and 4-(2-aminoethyl) benzenesulfonyl fluoride AEBSF (Sigma-Aldrich Co., St. Louis, MO, USA). After centrifugation at 2000 g for 30 min at 4 °C, the supernatant was collected and kept frozen at −70 °C until use. Immunoglobulin levels in intestinal fluid were evaluated by enzyme-linked immunosorbent assay (ELISA) using goat anti-mouse IgA (M8769, Sigma-Aldrich Co.) and goat anti-mouse IgA horseradish peroxidase-conjugated (A4789, Sigma-Aldrich Co.). Color was developed with o-phenylenediamine (OPD, Sigma-Aldrich Co.) in phosphate-citrate buffer pH 5.0, stopped with 3N H_2_SO_4_, and read spectrophotometrically at 492 nm in an ELISA plate reader (Thermo Fisher Scientific Inc., Rockford, IL, USA). The concentrations of the immunoglobulin were determined using a purified mouse IgA standard (Southern Biotechnology Associates, Birmingham, UK). The results are expressed as µg sIgA/g intestinal content.

### 2.6. Histological and Morphometric Analysis of Mice Organs

At the end of experiment, control and probiotic-treated mice, challenged or not with *Salmonella*, were killed by cervical dislocation, and the liver and small intestine were removed. The distal small intestine was opened along the antimesenteric border and stretched on filter paper, prefixed with Bouin’s fluid containing 2.5% glacial acetic acid and rolled into a spiral with the mucosa facing inward to form a “Swiss roll” [30]. The material was routinely processed for paraffin embedding, and two consecutive 4-m-thick histological sections were obtained for hematoxylin-eosin staining and for IgA immunofluorescence technique in the small intestine. For morphometric examination, images were obtained using a micro analyzer program and the JVC TK-1270/RGB KS 300 Image Software Kontron Elektronick/Carl Zeiss image analyzer (Oberkohen, Germany). The overall architecture of the small intestine and liver tissues and aspects of mucosal or parenchyma damages were also evaluated by a single pathologist. A numerical value was attributed to the changes observed in the intestinal layers (mucosa and lamina propria) or in the liver (area of degenerative alterations of parenchyma and inflammatory infiltrate) and each animal received a score that was generated by a sum of all observed changes (maximum index 6) as described in [31]. Sections of the small intestine were evaluated by the following parameters graded 0 (normal) to 3 (severe injured): hyperemia, edema, hemorrhage, and neutrophil infiltrate to the intestine epithelium and lamina propria. Similarly, sections of the liver were evaluated by the parameters graded 0 (normal) to 3 (severe injured): area and degree of degeneracy of parenchyma structure and area of inflammatory infiltrate. Results were expressed as the median and standard deviation of the scores.

For quantitation of IgA^+^ cells area in small intestine by IgA immunofluorescence staining was used goat anti-mouse IgA (α-chain specific)-FITC antibody (F9384, Sigma-Aldrich). On average were measured 10 histological fields using 10x objective per animal. The results were expressed as mean and standard deviation of the ratio (in %) in the area of IgA^+^ cells/total area of the ileum in each image.

### 2.7. Salmonella Typhimurium Translocation

Bacteria translocation to liver and spleen of mice were determined 9 days after infection by *Salmonella*. After the sacrifice, the organs were aseptically collected, weighed, and macerated in sterile PBS (1:10, w/v). Serial tenfold dilutions were prepared and 100 μL aliquots were plated onto MacConkey agar (Difco, Sparks, MD, USA). Colonies were counted after incubation at 37 °C for 24 h.

### 2.8. Total RNA Isolation

Total RNA was isolated from the intestinal fragments described above using Trizol (Life Technologies Corp., Carlsbad, CA, USA), according to the manufacturer’s instructions. After the removal of RNAlater, phenol extraction was performed using a procedure adapted from [32]. Briefly, samples were disrupted (5% w/v) in lysis buffer (4 M guanidine thiocyanate, 25 mM sodium citrate (pH 7.0), 0.5% N-lauryl sarcosine and 0.1 M β-mercaptoethanol) with a blender (Waring Products, New Hartford, CT, USA). RNA was purified from 900 μL of lysates using 90 μL of 2 M sodium acetate (pH 4.0), 810 μL of phenol and 180 μL of 24:1 chloroform/isoamyl alcohol. The total RNA was submitted to electrophoresis on 1% agarose gel to evaluate the quality, before quantification by spectrophotometry in a NanoDrop system (Thermo Scientific Inc., Bremen, Germany). Only RNA samples with >200 µg/mL of RNA and an A_260_/A_280_ ratio between 1.7 and 2.1 were used. Genomic DNA was removed from 10 µg of the RNA using Turbo DNase I, according to the manufacturer’s instructions (Life Corp. Technologies, Grand Island, NY, USA).

### 2.9. Reverse Transcription

Total RNA was reverse transcribed in a 20 μL final volume from 1 µg total RNA (DNA-free) using High-capacity cDNA reverse transcription (Life Technologies Corp.), according to the manufacturer’s instructions, with 500 ng random primers and 1 U MultiScribe™ MuLV reverse transcriptase.

### 2.10. Real-Time RT-qPCR Analysis

The relative quantification of cytokine gene expression of interleukin 5 (IL-5), IL-6, IL-10, IL-12b, IL-17a, gamma interferon IFN-γ), transforming growth factor beta 1 (TGF-β1) and tumor necrosis factor alpha (TNF-α)) in the cDNA samples from intestinal tissue was done by real-time RT-qPCR. The total RNA was obtained as described above. Real-time RT-qPCR was performed using a SYBR Green PCR Master Mix 2× kit, according to the manufacturer’s protocol (Applied Biosystems, Foster City, CA, USA). Gene-specific primers for the cytokines and reference genes ACTB and GAPDH were used according to [33]. The reactions were performed according to the optimized parameters described in [28] using an ABI Prism 7900 HT sequence detection system and the analysis was conducted using Sequence Detection software Version 2.4 (Applied Biosystems). The expression levels in the saline control group and *Salmonella* control group were used as the calibration data in challenged and no-challenged groups, respectively. The results were expressed graphically using the means and standard deviations of the relative mRNA levels (RLmRNA) for each cytokine, which were normalized against the reference gene expression level.

### 2.11. Statistical Analysis

The results were analyzed using GraphPad Prism^®^ Version 5.0 (Graph Pad Software Inc., San Diego, CA, USA). The means, standard deviations and coefficients of variation were obtained for each dataset. Shapiro-Wilk normality test was done and the data considered non-parametric. Kruskal-Wallis one-way analysis of variance followed by pairwise multiple comparisons using Mann-Whitney U test was used. The results were considered significant at *p* < 0.05.

## 3. Results

*L. salivarius* (L38) and *L acidophilus* (L36) strains were previously isolated from bovine stool samples (unpublished data) and in *in vivo* experiments with germ-free mice they successfully colonized and changed the expression of cytokines in the gastrointestinal tract of gnotobiotic animals [28]. Herein, L38 and L36 bacteria were supplied as live suspensions to mice challenged with *Salmonella enterica* serovar Typhimurium to evaluate, on the 9th day post-infection, their effects on animal weight, spleen and liver size indexes, and small intestine architecture and histological aspects.

**Figure 1:**
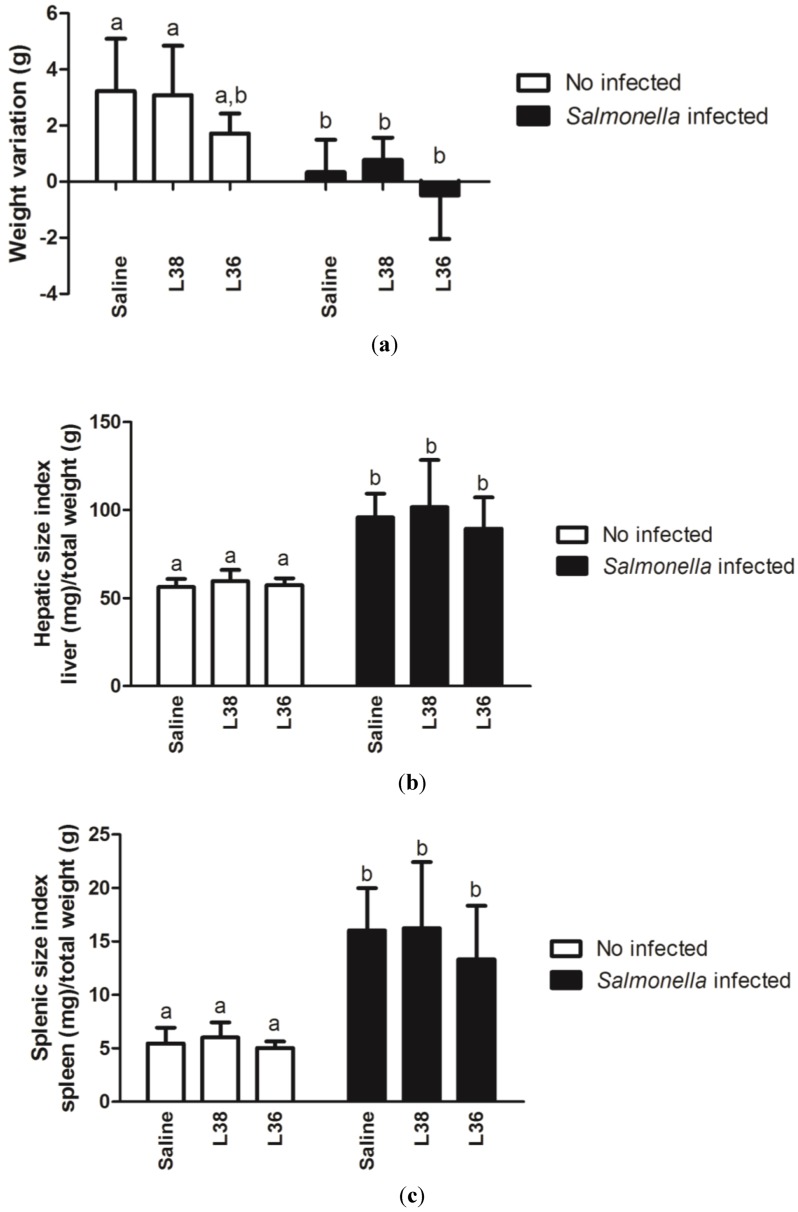
General health indicators of L38- and L36-pretreated mice on the 9th day post*-Salmonella* challenge. (**a**) Animal weight variation; (**b**) hepatic size index; (**c**) splenic size index. Results were expressed as mean of weight variation (g), hepatic size index (mg of spleen/g total weight), and splenic size index (mg of liver/g total weight) (*n* = 10 per group). The vertical bars indicate the standard deviation of means. Different letters over the bars indicate statistically significant differences between the experimental groups (*p* < 0.05).

Figure 1a shows the effects of the L38 or L36 strain gavage in the animal weight on the 9th day post*-Salmonella* challenge. Mice pre-treated with both strains did not show weight changes when compared to control animals (saline group). However, significant reduction in weight was seen in all groups of *Salmonella*-infected animals (*p* < 0.05). Figure 1b,c show the hepatic and splenic size indexes, respectively, of mice receiving both lactobacilli and challenged with *Salmonella*. Liver and spleen doubled and tripled in size, respectively, in the infected animals pre-treated or not with L38 or L36 strain. No probiotic strain had prejudicial effects on these general health indicators.

**Figure 2:**
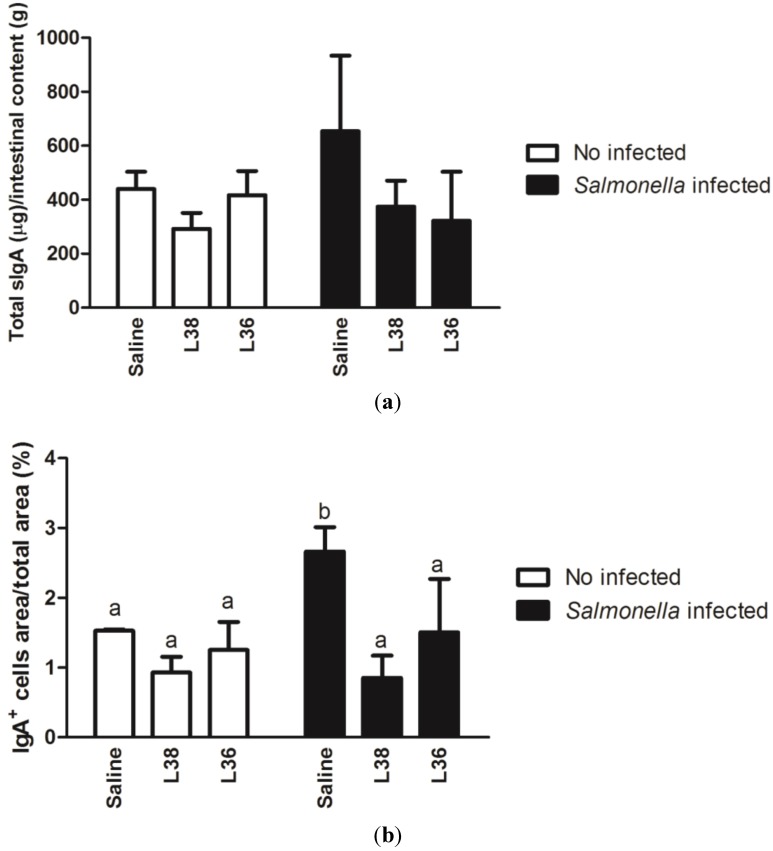
Production of secretory immunoglobulin A (sIgA) in the small intestine of L38- and L36-pretreated mice on the 9th day post*-Salmonella* challenge. (**a**) sIgA amount in the luminal content; (**b**) proportion of IgA producing (IgA^+^) cells area in the ileum. Results are expressed as mean of µg sIgA/g intestinal content and percentage of IgA^+^ cells area/total area (*n* = 10 per group). The vertical bars indicate the standard deviation of means. Different letters over the bars indicate statistically significant differences between the experimental groups (*p* < 0.05).

Next, we evaluated the secreted immunoglobulin A (sIgA) amounts in the intestinal content, and the proportion of IgA positive (IgA^+^) cells in the ileum of animals. Figure 2a shows the amount of sIgA (µg/g intestinal content) in animals receiving L38 and L36 strains on the 9th day post*-Salmonella* challenge. We observed a tendency for increasing sIgA levels with *Salmonella* infection compared to control animals, an effect that was reversed with *Lactobacillus* pretreatment (*p* < 0.05). A similar finding was seen when the proportion of IgA^+^ cells area in the ileum was determined (Figure 2b). Indeed, *Salmonella* infection increased significantly the proportion of IgA^+^ cells area in the ileum, which lowered to control levels with *Lactobacillus* administration (*p* < 0.05).

**Figure 3:**
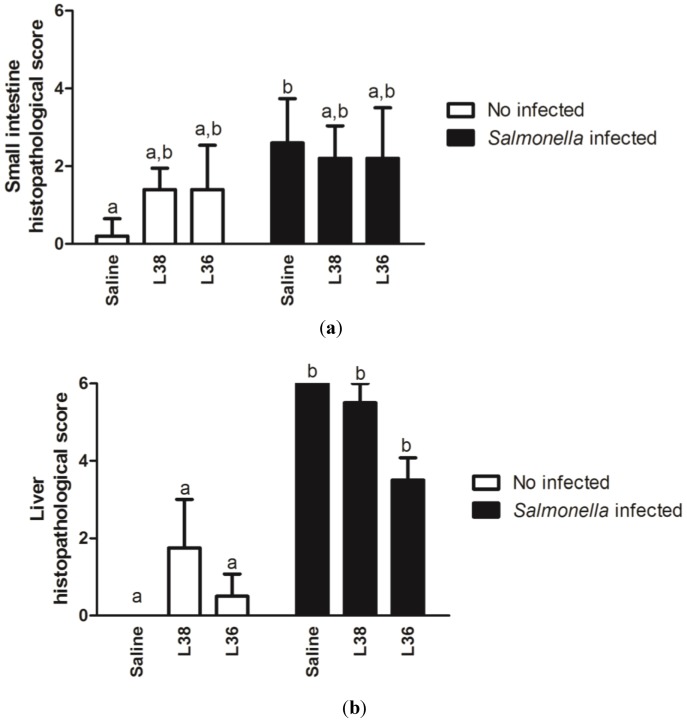
Histopathological scores of liver and small intestine of L38- and L36-treated mice on the 9th day post*-Salmonella* challenge. (**a**) Small intestine histopathological score and (**b**) Liver histopathological score. Results were expressed as mean histopathological scores according to the gradation 0 (normal) to 3 (severe) of tissue architecture damage (*n* = 10 per group). The vertical bars indicate the standard deviation of means. Different letters over the bars indicate statistically significant differences between the experimental groups (*p* < 0.05).

Control mice presented the small intestine with preserved mucosa and muscular wall, usual aspect of lamina propria, and continuous epithelial layer with evident brush border and globet cells. Small intestine of *Salmonella*-infected animals were diffusely and intensely affected showing extensive ulceration, irregular villous shortness and blunting, loss of enterocyte brush border, luminal sloughing of cellular debris, and lamina propria edema. Small intestine of animals treated with L38 and L36 strains did not show apparent changes when compared to the intestine of control animals. *Salmonella*-challenged animals pre-treated with L38 and L36 presented discrete changes such as small areas of brush border discontinuity and discrete and focal villous shortness and blunting. *Salmonella* infection in the colon has not been well characterized, and there were no typical lesions in infected animals. There was no statistical difference in small intestine histopathological scores among pre-treated mice with L38 and L36 no-*Salmonella*-challenged groups with respect to the group that received only saline (*p* > 0.05). Among all the infected groups was also not detected statistical difference (*p* > 0.05). However, there was a significant (*p* < 0.05) increase in small intestine histopathological scores in *Salmonella*-challenged groups compared with no-infected ones (Figure 3a). *Salmonella*-infected animals presented diffuse cell infiltrate that disrupted the normal lobular architecture of the liver and induced vacuolar degenerative changes, while both L38- and L36-treated and challenged animals better preserved the lobular architecture and hepatocytes aspects, despite the presence of parenchyma inflammatory cell infiltration in liver. However, there were no statistical differences (*p* > 0.05) in liver histopathological scores among the L38 and L36-treated groups and control one. Only significant increase (*p* < 0.05) in liver histopathological score was seen in challenged groups compared to no-challenged ones (Figure 3b).

**Figure 4:**
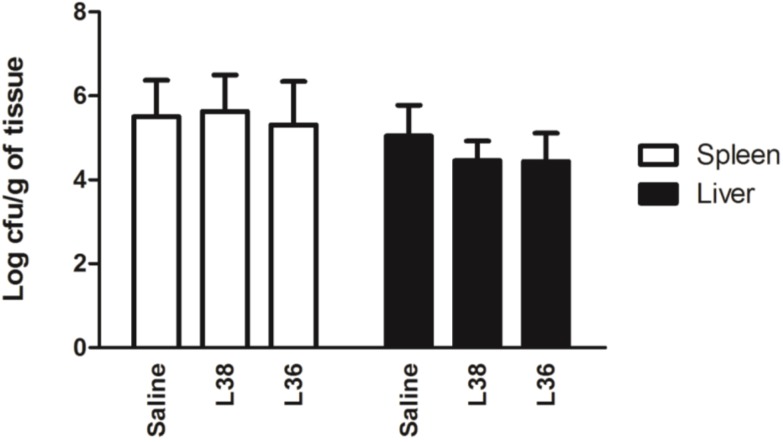
Translocation of *Salmonella* Typhimurium to liver and spleen of L38- and L36-treated mice on the 9th day post*-Salmonella* challenge. The bacterial counts are expressed as mean log cfu/g organ (*n* = 10 per group). The vertical bars indicate the standard deviation of means. No letters over the bars indicate no statistically differences between the experimental groups (*p* > 0.05).

The results in Figure 4 show *Salmonella* Typhimurium translocation to spleen and liver. Pre-treating mice with L38 or L36 strains did not alter the number of translocated *Salmonella* cells when compared with saline control (*p* > 0.05). *Salmonella* translocation was not detected in no-challenged animals.

**Figure 5:**
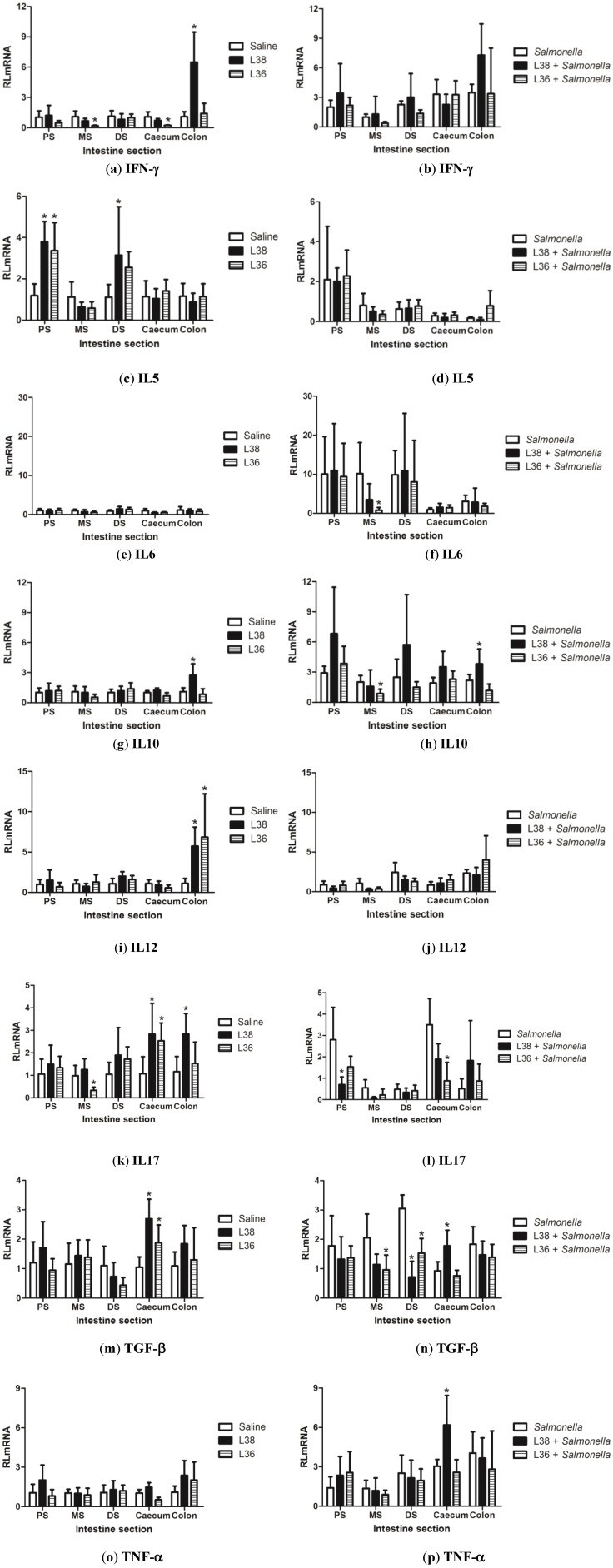
Relative gene expression of proinflammatory and regulatory cytokines in the intestine portions (PS: proximal segment, MS: middle segment, and DS: distal segment of small intestine, caecum and colon) of L38- and L36-treated mice on the 9th day post*-Salmonella* challenge. (**a**, **b**) IFN-γ; (**c**, **d**) IL5; (**e**, **f**) IL6; (**g**, **h**) IL10; (**i**, **j**) IL12; (**k**, **l**) IL17; (**m**, **n**) TGF-β; (**o**, **p**) TNF-α. Data were expressed as relative mean of mRNA amounts (RLmRNA) using as calibrator the expression level in saline control mice for the experimental groups (*n* = 10 per group). The vertical bars indicate the standard deviation of means. Asterisk over the bars indicate statistically significant differences between the experimental and control group (*p* < 0.05).

The relative quantification (RLmRNA) of the mRNA levels of genes encoding cytokines IL-5, IL-6, IL-10, IL-12b, IL-17a, IFN-γ, TGF-β1 and TNF-α was performed in different portions of the intestinal tract (small intestine: proximal, middle and distal segment; and large intestine: caecum and colon) of L38 and L36-pretreated mice challenged or not with *Salmonella*. The results obtained for L38 and L36-treated and saline-treated animals for no-challenged and challenged mice are shown in Figure 5. In non-challenged animals, L38 administration stimulated the mRNA expression levels of IL-5 in the proximal and distal segment of small intestine, IL-17a and TGF-β1 in the caecum, and IFN-γ, IL-10, IL-12b and IL-17a in the colon. L36-treated animals exhibited increased mRNA expression of IL-5 in the proximal segment of small intestine, TGF-β1 and IL-17a in the caecum and IL-12b in the colon, and a reduced expression of IL-17a in the middle segment of small intestine. In *Salmonella*-infected mice, pre-treatment with L38 strain reduced the expression of IL-17a and TGF-β1 in the proximal and distal segment of small intestine, respectively. On the other hand, L38 administration stimulated the mRNA expression levels of TNF-α and TGF-β1 in the caecum and IL-10 in the colon. L36-treated animals showed reduced levels of IL-6 and TGF-β1 in the middle segment of small intestine, IL-10 and TGF-β1 in the distal segment of small intestine, and IL-17a in the caecum.

## 4. Discussion

Using an experimental mouse model of salmonellosis, we aimed to characterize the safety and immune response modulation by *L. salivarius* (L38) and *L acidophilus* (L36) strains recently isolated from bovine. These lactobacilli showed *in vitro* putative probiotic features and could potentially substitute the traditional antibiotics commonly used in cattle rearing. These isolates showed success in colonizing the gut of germ-free mice, and had effects on cytokines profile expressed in the intestine of gnotobiotic animals [28].

Infectivity and pathogenicity are important components of safety study of new potentially probiotic strains [34]. Use of animal models is recommended as the gold standard for evaluating of the functional and safety features of newly isolated putative probiotics. Absence of macroscopic and microscopic changes in the intestine, spleen and liver, as well as general indicators of the health of L38 and L36-treated animals indicates that these bacteria are apparently safe. Degradation of the intestinal mucosa has been used as an early marker for pathogenicity of a new probiotic strain [1]. Other potentially probiotic strains that had been freshly isolated and had no history of safe use in humans or animals, showed similar results. *Lactobacillus rhamnosus* HN001, *L. acidophilus* HN017 and *Bifidobacterium lactis* HN019 administered to BALB/c mice did not cause changes in liver and spleen size indexes, histological features of liver, intestines and spleen, and other health parameters [35].

In no-challenged animals, L38 produced a diverse cytokine profile leading to increased mRNA levels of IL-5, IL-12b, IFN-γ, IL-17a, IL-10 and TGF-β1 in different portions of the intestine. Probiotics as *L. brevis* subsp. *coagulans* (Labre) and *Bifidobacterium lactis* HN019 cause increased production of IFN-γ [36]. TNF-α, IL-12 and IL-1β were higher produced in intestines of mice when treated with *L. reuteri* ML1 and *L. brevis* ML12 [13]. *L. casei*, *L. delbrueckii* subsp. *bulgaricus* and *L. acidophilus* induced rise in number of TNF-α and IFN-γ secretory cells in intestine [15]. Enhanced expression of TGF-β1 was observed *in vitro* in co-culture of *Lactobacillus johnsonii* and Caco-2 cells [37]. Together, TGF-β1 and IL-10 are main cytokines produced by T regulatory (T_reg_) cells of type T_r1_ that play important roles in regulating immune responses [38]. Differently, L36 treatment changed the expression of a less diverse cytokine panel in the intestinal mucosa (reduction of IL-17a in small intestine and increase of TGF-β1and IL-12b in large intestine) in no-challenged animals. Commensal bacteria appear to down-regulate IL-17a production by T_H_17 cells via up-regulation of IL-25, which selectively suppresses IL-23 secretion by intestinal dendritic cells with resultant reduction of IL-17a and inflammation [39]. Reduction of IL-17a production in intestine has been associated with relief of symptoms related to inflammatory bowel diseases, such as ulcerative colitis [40].

Due to the diverse profile of cytokines expressed in L38-treated mice throughout the intestines, it is possible to speculate that L38 induce a balance between T_H_ and T_reg_ responses in intestinal mucosa. *L. rhamnosus* GG has been associated with relief of symptoms and prevention of allergy for maintaining of homeostatic balance between T_reg_/T_H_1/T_H_2 cells [41,42]. *L. rhamnosus* HN001, normally considered a strong inducer of IFN-γ production, was administered orally to mice for an allergic sensitization (which generates T_H_2 responses), and in these animals augmented the secretion of IL-4 and IL-5 [43]. Mucosal immune system normally keeps a state that promotes tolerance to itself and IgA production, showing a slight deviation in T_H_1 over T_H_2 polarization, which characterizes a physiologically healthy state. Therefore, an adequate balance between pro-inflammatory (T_H_1, T_H_2 and T_H_17) and regulatory responses (T_reg_) is important to preserve mucosa healthy [40]. Thus, probiotic supplementation can correct dysbiosis and restore intestinal homeostasis by immunomodulatory mechanisms induced by these bacteria [44]. Therefore, L38 shown an interesting candidate as a probiotic strain to induce the expression of a set of anti-inflammatory (IL-10 and TGF-β1) and pro-inflammatory cytokines (IL-5, IL-12, IL-17a, IFN-γ) and may contribute to a proper balance between different subtypes of polarized CD4^+^ T cells in intestinal mucosa.

Absence of significant change in general health parameters (weight gain, liver and spleen size indexes) and small intestine and liver histological scores in all *Salmonella* infected mice despite pre-treatment with L38 or L36 strain were strong indications of no-protection by these isolates against the infection. In addition, reduction of *Salmonella* translocation to liver and spleen in L38 or L36-treated animals was negligible. Cytokine expression data in intestinal mucosa showed that L38 or L36 treatment in challenged animals reduced the expression of important cytokines need for fight the infection, such as IL-6 and IL-17a, and did not increase the expression of other cytokines involved in protection against infection, such as IL-12b and IFN-γ. Activation of IL12b:IL18➔ IFN-γ axis is one of most important mechanisms in the fight against *Salmonella* infection [45]. Lipopolysaccharide (LPS) and certain lipoproteins of the cell wall of *Salmonella* induce a strong local inflammatory response in intestinal tissue leading to increase of TNF-α, IL-1, IL-6, IL-12b and IL-18 secretion [46]. IFN-γ production is a major milestone in immune responses against Salmonella and its increased production is often associated with protection against infection [47]. In recent years, it has been reported that responses involving IL-17a/T_H_17 appear to complement the response of IFN-γ/T_H_1 axis that is characteristic of the combat against infection caused by *Salmonella* [48,49]. Therefore, according the data is safe to say that L38 and L36 treatments did not provide protection against *Salmonella* Typhimurium infection. Probiotics containing *L. acidophilus* and *B. bifidum*, and a commercial yoghurt fermented with *L. acidophilus* and *Bifidobacterium spp.*, have also failed to protect animals against infection caused by *Salmonella* [50].

Significant expression of IL-10 cytokine was observed in the colon of no *Salmonella*-challenged mice, and in the initial and distal portion of small intestine and caecum of challenged animals, pre-treated with L38 strain. Other probiotic strains have the same effect. *L delbrueckii* subsp. *bulgaricus* and *L. casei* induced increase of IL10^+^ cells in small intestine of mice [15]. Commensal bacteria have also been implicated in the super-expression of IL-10, with experimental evidence that IL-10 secretion increases following stimulation with *Bifidobacterium* sp. [51]. Many studies indicate that rise of IL-10 production in intestinal mucosa is correlated with protection in ulcerative colitis chemically induced in mice [52,53] possibly as a result of clonal expansion of T_reg_ subsets in intestinal mucosa [54]. Probiotic VSL#3 decreased colitis severity in an inflammatory bowel disease animal model due to an increased production of IL-10 by generating large numbers of T_reg_ cells in lamina propria [55]. Given this, L38 appears to be a promising candidate to be evaluated in animal models of inflammatory bowel disease.

To access the humoral immune response locally at the mouse intestine, we quantified the total sIgA secreted to intestinal lumen and the proportion of IgA^+^ secreting cells in the tissue in response to the administration of saline or *L. salivarius* L38 and *L. acidophilus* L36 suspensions. No-differences were seen between these animals but sIgA levels tended to increase on the 9th day after infection with *Salmonella* in accordance with the significant enhancement of IgA^+^ cells at the ileum. Probably, this increased proportion of IgA^+^ secreting cells is related to stimulation by the bacterial lipopolysaccharide (LPS) [56]. Secreted IgA is an important effector molecule of mucosal immunity, acting as the first barrier against pathogen infections [57]. In salmonellosis, it has been demonstrated that luminal IgA and IgM could block *Salmonella* penetration of tissues, probably inhibiting the binding of bacteria to epithelial cells and M cells [58]. This increased of sIgA appears to be supported by high levels of production of IL-6 and TGF-β1 expressed in the small intestine of challenged controls animals (data not shown) that likely contribute to the isotype switching of immature B cells to sIgA producing plasmatic cells [59]. Mice fed with the putative probiotic strains L38 and L36 and challenged with *Salmonella* showed similar sIgA levels and proportion of IgA^+^ secreting cells in the ileum as the non-infected animals, with a significant reduction observed in relation to *Salmonella*-infected mice without probiotic treatment. These results show that the administration of these probiotic bacteria down regulated the production of IgA antibody in the mouse model of salmonellosis.

Other probiotics, *L. acidophilus* NCDC14 and *L. casei* NCDC19, have been shown to increase sIgA synthesis and secretion in the intestinal mucosa, a fact that may explain the adjuvant effects shown by these bacteria in different experimental models [60,61]. However, there is some controversy on whether or not the beneficial effects are due to the increased levels of IgA. This may be explained by the kinetics of IgA production during the course of *Salmonella* infection (and therefore reflecting the time at which IgA dosage was done), mouse lineage, and initial inoculum dose of *Salmonella* [62]. Despite this, both sIgA secreted levels and proportion of IgA^+^ cells analyses performed produced similar results, reinforcing the sensitivity of both indexes to address IgA in mouse intestinal mucosa. Some traditional probiotic strains such as *L. acidophilus* La1 and *L. casei* Shirota does not alter sIgA level in intestinal mucosa [63,64]. Because no significant differences in sIgA secretion and proportion of IgA^+^ cells between either L38 or L36 strain were found, it is plausible to assume that these parameters are not good immunological markers to select putative probiotics, corroborating previous findings [65,66].

## 5. Conclusions

Our findings constitute the first report on the immunomodulatory effects in conventional animals of putative probiotics isolated from bovines, *L. salivarius* L38 and *L acidophilus* L36. Ultimately, the idea is to include microbials with probiotic features as natural food supplements to replace the antibiotics commonly added to feed or water, a practice shown to have dangerous impacts on human and livestock health. We believe the study model presented here will help future research on the use of probiotics in cattle rearing to be more easily pursued.
